# Gut Microbiota in Celiac Disease: Is There Any Role for Probiotics?

**DOI:** 10.3389/fimmu.2020.00957

**Published:** 2020-05-15

**Authors:** Francesco Pecora, Federica Persico, Pierpacifico Gismondi, Fabiola Fornaroli, Silvia Iuliano, Gian Luigi de'Angelis, Susanna Esposito

**Affiliations:** ^1^Department of Medicine and Surgery, Pediatric Clinic, Pietro Barilla Children's Hospital, University of Parma, Parma, Italy; ^2^Unit of Gastroenterology and Digestive Endoscopy, Department of Medicine and Surgery, University of Parma, Parma, Italy

**Keywords:** celiac disease, dysbiosis, gut microbiota, microbiome, probiotics

## Abstract

Celiac disease (CD) is an immune-mediated disorder initiated by the ingestion of gluten in genetically predisposed individuals. Recent data shows that changes in the gut microbiome composition and function are linked with chronic inflammatory diseases; this might also be the case for CD. The main aim of this manuscript is to discuss our present knowledge of the relationships between gut microbiota alterations and CD and to understand if there is any role for probiotics in CD therapy. PubMed was used to search for all of the studies published from November 2009 to November 2019 using key words such as “Celiac Disease” and “Microbiota” (306 articles), “Celiac Disease” and “Gastrointestinal Microbiome” (139), and “Probiotics” and “Celiac Disease” (97 articles). The search was limited to articles published in English that provided evidence-based data. Literature analysis showed that the gut microbiota has a well-established role in gluten metabolism, in modulating the immune response and in regulating the permeability of the intestinal barrier. Promising studies suggest a possible role of probiotics in treating and/or preventing CD. Nevertheless, human trials on the subject are still scarce and lack homogeneity. A possible role was documented for probiotics in improving CD-related symptoms, modulating the peripheral immune response and altering the fecal microbiota, although the results were not consistent in all of the studies. No evidence was found that probiotic administration might prevent CD onset. Knowledge of the role of intestinal bacteria in the development of CD opens new possibilities for its treatment through probiotic administration, even though further studies are needed to better clarify whether probiotics can help treat or prevent the disease and to define which probiotics to use, at what dose and for how long.

## Introduction

Celiac disease (CD) is an immune-mediated disorder initiated by the ingestion of gluten in genetically predisposed individuals (1, 2). In patients with CD, immune responses to gliadin fractions stimulate an inflammatory response. This reaction is mediated by the innate and adaptive immunity (3). The interaction between gliadin peptides and the G protein-coupled receptor CXCR3 on enterocytes triggers the release of zonulin, a potent intestinal barrier function modulator. Consequently, gliadin peptides translocate into the lamina propria and activate the immune response (4). In the lamina propria, intestinal tissue transglutaminase (tTG) reacts with gliadin peptides to deamidate them to negatively charged glutamic acid residues that are highly immunogenic. After tTG-induced deamidation, gliadin peptides activate the humoral immune response with antibodies against gliadin and tTG and to the production of pro-inflammatory cytokines, such as interferon gamma (IFNγ), interleukin-17 (IL-17) and tumor necrosis factor-alpha (TNFα) (5).

Many researches have observed that different functions of macrophages, dendritic cells and neutrophils, which are an essential part of the innate immune system, are influenced by the microbiota (6, 7). Gliadin peptides also stimulate an innate immune response in the intestinal epithelium that is characterized by increased expression of IL-15 by enterocytes, resulting in the activation of intra-epithelial lymphocytes expressing the activating receptor NK-G2D, a natural killer cell marker (8, 9).

CD does not develop unless a person has alleles that encode HLA-DQ2 or HLA-DQ8 proteins, products of two of the HLA genes. However, HLA-DQ2 and HLA-DQ8 haplotypes are not specific for CD because many people, most of whom do not have CD, carry these alleles; thus, the DQ2 and DQ8 haplotypes are necessary but not sufficient for the development of CD (10, 11). Furthermore, with the introduction of GWAS (genome-wide association studies), an additional 39 non-HLA regions of susceptibility have been associated with CD development (12, 13). Several studies have identified more genes associated with CD that are involved in immune function or related to defects in intestinal permeability or with bacterial colonization and sensing (14, 15).

The role of an environmental influence in CD pathogenesis is supported by the facts that (a) HLA and non-HLA genes explain only 55% of disease susceptibility, (b) there is a lack of 100% concordance of CD in monozygotic twins and (c) the incidence of this condition is increasing (16–18). Based on more recent epidemiological data, loss of gluten tolerance may occur at the time of its introduction into the diet or at any time in life, with several different intestinal and extra-intestinal symptoms (19–22). These findings suggest that other environmental factors must play a role in CD development. Indeed, environmental factors that influence the composition of the human gut microbiota, such as birth gestational age, type of delivery, intestinal infections and antibiotic exposure, have been associated with the risk of developing CD (23–26).

Gut bacteria are key regulators of digestion along the gastrointestinal tract and have a relevant impact in the synthesis of many nutrients and metabolites (27–29). Furthermore, the gut microbiota has a crucial immune function, inhibiting bacterial growth and maintaining intestinal epithelial integrity (30). Moreover, growing evidence has shown a critical role for commensal bacteria and their products in influencing the development, homeostasis, and function of innate and adaptive immune cells (31, 32). Recent data support the hypothesis that changes in the gut microbiome composition and function are linked with chronic inflammatory diseases; this might also be the case for CD (33). Although a gluten-free diet (GFD) influences the gut microbiota composition and diversity and thus represents a confounding factor, several studies support the hypothesis that the microbiota plays a role in the pathogenesis, clinical manifestation and risk of developing CD (34). Moreover, it has been reported that soluble CD14 (sCD14, i.e., an indicator of innate immune cell activation in response to mucosal translocation of Gram-negative bacteria) is increased in untreated patients with CD, probably because of translocation of commensal intestinal bacteria (35, 36). Finally, patients with persistent symptoms on a long-term GFD have an altered microbiota composition (37).

The evidence of intestinal dysbiosis in CD, together with the role of the gut microbiota in regulating the immune response, opens up the possibility of finding new therapeutic approaches by modulating the intestinal microbiota with the use of probiotics. The main aim of this manuscript is to discuss our present knowledge of the relationships between gut microbiota alterations and CD and to understand if there is any role for probiotics in CD therapy. PubMed was used to search for all of the studies published from November 2009 to November 2019 using key words such as “Celiac Disease” and “Microbiota” (306 articles), “Celiac Disease” and “Gastrointestinal Microbiome” (139 articles), and “Probiotics” and “Celiac Disease” (97 articles). The search was limited to articles published in English that provided evidence-based data.

## Gut Microbiota and Environmental Factors

Each healthy individual has a unique gut microbiota. Core native microbiota are shaped in early life (i.e., in the first 36 months of age) by gut maturation, which is strongly influenced by environmental factors such birth gestational age, type of delivery, method of milk feeding, weaning period, lifestyle, and dietary and cultural habits (38). When a child is 2–3 years old, a relative stability in gut microbiota composition has been documented (33, 39). Gut microbiota are represented by several species of microorganisms, including bacteria, yeast, and viruses. The two major bacterial phyla are *Firmicute*s and *Bacteroidetes*, which are 90% of the whole gut microbiota (40). The *Firmicutes* phylum is composed of ≥ 200 different genera, and *Clostridium* genera are 95% of the *Firmicutes* phyla. Bacteroidetes consists of predominant genera such as *Bacteroides* and *Prevotella*. *Actinobacteria, Proteobacteria, Fusobacteria*, and *Verrucomicrobia* are the next most numerous phyla, which are described in a “healthy gut microbiota composition” (33, 40).

It has been observed that the HLA-DQ genotype can influence early gut microbiota composition (41). Several studies have demonstrated that the genotype of infants at family risk of developing CD, carrying the HLA-DQ2 haplotypes, influences the early gut microbiota composition. Olivares et al. (42) reported that infants with a high genetic risk have significantly higher proportions of *Firmicutes* and *Proteobacteria* and lower proportions of *Actinobacteria* and *Bifidobacteria* compared with low-risk infants. In a study including 164 healthy new-borns with ≥one first-degree relative with CD, De Palma et al. (43) showed that milk-feeding type in conjunction with HLA-DQ genotype has an impact in in establishing infants' gut microbiota.

Diet represents another key regulator of microbiome development and homeostasis. Although recent data have shown that breast-feeding has no protective effect on the development of CD, studies have reported that genotype-related differences in microbiota composition are reduced by breast-feeding (44). Moreover, human milk oligosaccharides enhance overall barrier integrity by making enterocytes less vulnerable to bacterial-induced innate immunity (45).

Some observational studies have also shown that elective cesarean delivery is linked with an increased risk of CD onset during pediatric age, suggesting the impact of dysbiosis during early life. The gut microbiota of children born by elective cesarean section, compared to vaginally delivered infants' microbiota, has reduced microbial diversity and fewer *Bifidobacterium* species (46, 47). According to some studies, antibiotic exposure during the first year of life has been linked with intestinal dysbiosis, reduced fecal microbial diversity, and early onset of CD (48, 49). However, there are other studies that did not confirm this result (50, 51), and a recent meta-analysis found no evidence of an association between prenatal or postnatal antibiotic exposure and CD (52).

Although it is currently recognized how these environmental factors influence the composition of the intestinal microbiota, there are no longitudinal studies that have defined whether and how the gut microbiota plays a role in the development of CD. A large, ongoing, multi-center, prospective longitudinal study called CDGEMM (Celiac Disease, Genomic, Environmental, Microbiome, and Metabolomic Study) has the goal of identifying and validating specific microbiome and metabolomic profiles able to predict loss of tolerance in children genetically at risk of autoimmunity (53).

## Gut Microbiota and Implications in Celiac Disease (CD) Pathogenesis

The gut microbiota present in the human colon participates in gluten metabolism. *Lactobacilli* and *Bifidobacterium* spp. may play a role in the breakdown of gluten and its peptides to modify their immunogenic potential (54, 55). Caminero et al. (56) demonstrated that opportunistic pathogens and core gut commensals produce distinct breakdown patterns of gluten with increased or decreased immunogenicity that could influence autoimmune risk. In particular, it has been demonstrated that *Lactobacilli* can detoxify gliadin peptides after partial digestion by human proteases; additionally, immunogenic peptides produced by *Pseudomonas aeruginosa* proteases are also further degraded and rendered less immunogenic in the presence of *Lactobacillus*. These findings on the gluten-processing activities of specific microbial strains could suggest the use of probiotics as complementary therapy for CD.

Changes in the gut microbiota composition could also play a role in altering the intestinal barrier and increasing epithelial permeability (57). Disassembly of zonulin, that is involved in tight junctions, has a pathogenic role in increasing the intestinal permeability present in patients with CD. Some studies have reported that dysbiosis is associated with increasing zonulin release, disrupting tight junctions, and enhancing the entry of incompletely digested gliadin peptides into the lamina propria (4, 58). In addition, it is acknowledged that the gut microbiota has an important role in the regulation of host metabolism and immunity (59). Moreover, a recent study has shown how gut microbiota and their metabolites enhance the risk of developing autoimmunity through epigenetic processes (60). To find a microbial agent for disease immunomodulation, *Bifidobacteria* and *Lactobacilli* are the most studied strains. *Bifidobacteria* strains have been described to play a role in reducing the epithelial permeability triggered by gluten (61), in downregulating the Th1 pathway typical of CD (62), and in decreasing jejunal architecture damage (63). Furthermore, it has been reported that *Escherichia coli* could have a protective effect on gut barrier function (64) and that *Lactobacilli* strains have immunomodulatory properties (65).

In recent years, several cross-sectional studies have evaluated fecal, salivary, and duodenal microbiota associated with CD. Patients with CD show a decrease in beneficial species (*Lactobacillus* and *Bifidobacterium*) and an increase in those potentially pathogenic (*Bacteroides* and *E. coli*) in comparison with healthy subjects (66). In particular, some studies have reported that *Bifidobacterium* spp., *Bifidobacterium longum, Clostridium histolyticum, C. lituseburense*, and *Faecalibacterium prausnitzii* group proportions are less abundant in untreated patients with CD than in healthy controls (67–69). Ou Gangwei et al. (70) have shown that rod-shaped bacteria represented a significant fraction of the proximal small intestine microbiota in children with CD during the so-called Swedish epidemics. These bacteria present in the epithelial lining of the small intestine were observed in children with CD but not in controls. Another study analyzed the mucosa-associated microbiota of 20 children with CD, before and after a GFD regimen, and of 10 controls, with evidence of a peculiar microbial profile and a significantly higher biodiversity in the duodenal mucosa of patients with CD (71). Furthermore, Di Cagno et al. (72) demonstrated that a GFD lasting at least 2 years did not completely restore the microbiota of children with CD.

Wacklin et al. (34, 73) have shown microbiota alterations, particularly in subjects with persistent symptoms despite adherence to a long-term GFD or associated with gastrointestinal symptoms but not with dermatitis herpetiformis. These studies highlight that the alterations in gut microbiota are more pronounced in the active phase of CD, suggesting that perturbations in the interaction between the host and the microbiota could influence CD manifestation and evolution. However, the abovementioned CDGEMM study could help to understand the role that the gut microbiome show in the early steps involved in the pathogenesis of CD (53).

## Probiotic Supplementation in Celiac Disease (CD)

Probiotics have shown the ability to hydrolyse immunogenic gluten peptides (56, 74–77), thus reducing their immunogenicity. Moreover, intestinal bacteria have also been implicated in modulating the immune response, directing the correct differentiation of anti-inflammatory Treg cells. In patients with CD who underwent an oral wheat challenge, circulating T cells were collected, showing an increase in both effector T cells and Treg FOXP3+ cells (86); however, these FOXP3+ T cells were found to have a significantly reduced suppressive function. *Serena* and colleagues suggested that this impairment may be related to the presence of an alternatively spliced isoform of FOXP3 and hypothesized that the intestinal microenvironment may have an impact in modulating this alternative splicing (60). Furthermore, studies suggest that the composition of the intestinal microbiota can affect the permeability of the intestinal mucosa (87, 88). In addition, probiotics would encourage the intestinal microbiome to improve the production of short chain fatty acids such as butyrate that can have profound effects in modulating proinflammatory activities in the colonic gut and in inducing health effects on the colonic epithelia (74).

Considering this established role of microbiota in gluten metabolism and in modulating the gut immune response, the manipulation of the microbiome via probiotic administration opens new possibilities for the treatment of CD and its related symptoms. Reports on animal models have shown promising effects of probiotics on CD. *Bifidobacterium longum* CECT 7347 has been found to decrease the production of inflammatory cytokines and CD 4+ T cells in rats (63) as well as ameliorate gliadin-induced enteropathy (55). *Saccharomyces boulardii* KK1 oral administration improved enteropathy and decreased epithelial cell CD71 expression and local cytokine production in gluten-sensitized mice (89). Administration of *Lactobacillus casei* was found to be effective in rescuing the normal mucosal architecture in a mouse model of gliadin-induced villous damage (65). *Bifidobacterium breve* prevented intestinal inflammation through the induction of intestinal IL-10-producing Th1 cells (90) and ameliorated DSS-induced colitis symptoms in mouse models as well as modulated T cell polarization toward Th2 and Tregs both *in vitro* and *in vivo* (91). In a recent study, *Orlando* and colleagues found that *Lactobacillus rhamnosus* GG administration to rats could protect the intestinal mucosa from gliadin peptide-induced damage (92).

However, despite a number of promising *in vitro* and *in vivo* animal studies on probiotic use, data from human trials are still scarce and lack homogeneity. We reviewed studies on human patients with CD published from January 2009 to December 2019 regarding the efficacy and safety of probiotic supplementation (Table 1). Although no safety issue was observed in patients with CD treated with probiotics, a few studies have shown that probiotic administration might be beneficial in improving CD-related symptoms. Smecuol et al. (78) randomized patients with CD to receive either the *Bifidobacterium Infantis Natren life start* strain *or* placebo and found a significant improvement in gastrointestinal symptoms in the group receiving probiotic, although they were not following a strict GFT. Changes in intestinal microbiota were also documented in a 2016 study from Quagliarello et al. (81) who analyzed microbial DNA extracted from the feces of 40 pediatric patients with CD before and after probiotic treatment, evidencing an increase in *Actinobacteria* as well as a re-establishment of the physiological *Firmicutes/Bacteroidetes* ratio. Similar results were obtained by Primec et al. (84) in a 2019 double-blind placebo-controlled study of 40 children with CD who were randomized to receive either probiotics or placebo for 3 months. In contrast, Harnett et al. (80) found no differences in the fecal microbiota counts or in the severity of the symptoms between the group receiving probiotic and the placebo group. Lastly, it has been documented that probiotic treatment can modulate immunological parameters such as TNF-α and peripheral T lymphocytes. Klemenak et al. (79) in a double-blinded, randomized, placebo-controlled trial, showed the positive effect of *B. breve* strain administration in decreasing the production of the pro-inflammatory cytokine TNF-α in children with CD on a gluten-free diet. The administration of *Bifidobacterium longum CECT 7347* was also found to significantly decrease peripheral CD3+ T lymphocytes as well as cause a slight decrease in TNF- α (79). Daily oral administration of *L. plantarum* HEAL9 and *L. paracasei* 8700:2 for 6 months was related to changes in the immune response in 78 children with celiac disease autoimmunity in a 2019 study from Hakansson et al. (85) interestingly, the difference in most lymphocyte subsets found in the placebo group was similar to that found in patients with active celiac disease, indicating a progression of disease development that was not observed in the probiotic group. Two studies were conducted to investigate the ability of probiotics to prevent CD onset. The first, from *Savilahti* and colleagues, analyzed data taken from a trial on primary allergy prevention, including 1,223 babies treated with probiotics until the age of 6 months vs. placebo, and found no difference in the risk of developing CD during the 13-year follow-up (82). Later, Uusitalo et al. (83) conducted a multi-center study following over 6,000 genetically susceptible children for a median period of 8.7 years and found that probiotic administration did not change the risk of developing CD.

**Table 1:** Studies on human patients with celiac disease (CD) regarding the efficacy of probiotic supplementation.

**Study**	**Author and Reference**	**Methods**	**Type of Probiotics**	**Results**
Exploratory, randomized, double-blind, placebo-controlled, study on the effects of *Bifidobacterium infantis* Natren life start strain super strain in active celiac disease	Smecuol et al. (78)	22 patients with CD were given either probiotics or placebo for 3 weeks while not following a strict GFD.	*Bifidobacterium infantis* NLS super strain	The abnormal baseline intestinal permeability was not significantly affected by the treatment. Symptoms measured by the GSRS questionnaire were significantly improved in the group receiving probiotics (*P* = 0.0035 for indigestion; *P* = 0.0483 for constipation).
Administration of *Bifidobacterium breve* decreases the production of TNF-alpha in children with celiac disease	Klemenak et al. (79)	46 children with CD on a GFD randomized into two groups, one receiving probiotics and one receiving placebo for 3 months.	*Bifidobacterium breve* BR03 and *B. breve* B632	TNF-alpha levels significantly decreased from baseline in the probiotics group at the end of the 3 months (*p* = 0.020). On follow-up, 3 months after receiving probiotics, TNF-α levels increased again.
Probiotics and the microbiome in celiac disease: a randomized controlled trial	Harnett et al. (80)	A multi-center RCT conducted in Australia in 2011 on a group of 45 people with only partial response to GFD. Participants took 5 drops of VSL# twice daily for 12 weeks vs. controls taking 5 placebo drops.	VSL#3	No statistically significant changes in the fecal microbiota nor clinically significant improvement in symptoms was observed between the 2 groups.
Effect of *Bifidobacterium breve* on the intestinal microbiota of coeliac children on a gluten-free diet: A pilot study	Quagliarello et al. (81)	40 children with CD on a GFD were administered either two B. breve strains or placebo for 3 months. Microbial DNA was extracted from feces before and after treatment.	*Bifidobacterium breve* B632 and BR03 strains	A significant increase in Actinobacteria was found as well as a re-establishment of the physiological Firmicutes/Bacteroidetes ratio (*p* <0.01).
Celiac disease by the age of 13 years is not associated with probiotics administration in infancy	Savilahti et al. (82)	Data were taken from a trial on primary allergy prevention including 1223 babies with a high risk for allergy. Probiotics vs. placebo were given to mothers for 4 weeks before delivery and to infants until the age of 6 months.	*Lactobacillus rhamnosus* GG, *L. rhamnosus* LC 705, *Bifidobacterium breve* Bb99, *Propionibacterium freudenreichi* spp.	Probiotics administration did not affect the risk of developing CD during the 13-year follow-up.
Early probiotics supplementation and the risk of celiac disease in children at genetic risk	Uusitalo et al. (83)	Multi-center study following 6520 genetically susceptible children for a median period of 8.7 years, recording probiotics use by 1 year of age.	Various, mainly *Lactobacillus reuteri* and *L. rhamnosus*	Exposure to probiotics was not associated with a different risk of developing either celiac disease autoimmunity or celiac disease.
Clinical intervention using Bifidobacterium strains in celiac disease children reveals novel microbial modulators of TNF-α and short-chain fatty acids	Primec et al. (84)	Double-blind, placebo-controlled study of 40 children with CD who received either probiotics or placebo for 3 months.	*B. breve* BR03 (DSM 16604) and *B. breve* B632 (DSM 24706)	The Firmicutes/Bacteroides ratio was re-established*. Verrucomicrobia, Parcubacteria* and some yet unknown phyla, which may be involved in the disease, were highlighted, as indicated by a strong correlation to TNF-α. Modulating the production of short-chain fatty acids could play a role in restoring the microbiome.
Effects of *L. plantarum and L. paracasei* on the peripheral immune response in children with CD autoimmunity: a RCT	Hakansson et al. (85)	78 children with celiac disease autoimmunity received either probiotics or placebo for 6 months. Phenotyping of peripheral blood lymphocytes was conducted, and tTG was measured before and after treatment.	*L. plantarum* HEAL9 and *L. paracasei* 8700:2	Different subtypes of peripheral lymphocytes were found in the probiotics groups vs. placebo group. The median levels of IgA-tTG decreased more significantly over time in the probiotic (*p* = 0.013) than in the placebo (*p* = 0.043) group.

## Conclusions

Although the association between alterations in the gut microbiota and the development of CD has been demonstrated, a definite microbial signature and the exact role of dysbiosis in CD pathogenesis are not recognized. Further human studies will be needed to reach a definitive conclusion on the role of probiotics in CD using standardized probiotic formulations, dosages and periods of treatments, as well as homogeneous patient groups. Currently published data suggest the efficacy and safety of probiotic supplementation in improving CD-related symptoms (78), as well as documenting the ability of some probiotics to alter the fecal microbiota and decrease pro-inflammatory parameters such as TNF-α levels or peripheral CD3+ T lymphocyte counts (79, 81). Which probiotics are more effective, at what dose and how long they should be administered are yet to be definitively clarified. However, the encouraging data on *in vitro* and *in vivo* studies, as well as the knowledge of the mechanisms through which intestinal bacteria modulate the development of the disease, prompted us to continue the studies to achieve a better understanding of the possible role of probiotics in treating and/or preventing CD.

## Author Contributions

FPec and FPer wrote the first draft of the manuscript. PG, FF, and SI performed the literature analysis and revised the first draft. GA and SE critically revised the text and made substantial scientific contributions. All authors approved the final version of the manuscript.

## Conflict of Interest

The authors declare that the research was conducted in the absence of any commercial or financial relationships that could be construed as a potential conflict of interest.
